# Neurogenin3 phosphorylation controls reprogramming efficiency of pancreatic ductal organoids into endocrine cells

**DOI:** 10.1038/s41598-018-33838-5

**Published:** 2018-10-18

**Authors:** Roberta Azzarelli, Steffen Rulands, Sonia Nestorowa, John Davies, Sara Campinoti, Sébastien Gillotin, Paola Bonfanti, Berthold Göttgens, Meritxell Huch, Benjamin Simons, Anna Philpott

**Affiliations:** 1 Department of Oncology, University of Cambridge, Hutchison/MRC Research Centre, Hills Road, Cambridge, CB2 0XZ UK; 2 Wellcome - Medical Research Council Cambridge Stem Cell Institute, University of Cambridge, Tennis Court Road, Cambridge, CB2 1QR UK; 3 Cavendish Laboratory, Department of Physics, University of Cambridge, Cambridge, CB3 0HE UK; 4 Max Planck Institute for the Physics of Complex Systems, Nöthnitzer Str. 38, 01187 Dresden, Germany; 5 Center for Systems Biology Dresden, Pfotenhauer Str. 108, 01307 Dresden, Germany; 6 Department of Haematology, Cambridge Institute for Medical Research, Hills Road, Cambridge, CB2 0XY UK; 7 The Francis Crick Institute, 1 Midland Road, London, NW1 1AT UK; 8 UCL Great Ormond Street Institute of Child Health, 30 Guilford Street, London, WC1N 1EH UK; 9 The Wellcome/Cancer Research UK Gurdon Institute, University of Cambridge, Tennis Court Road, Cambridge, CB2 1QN UK

## Abstract

β-cell replacement has been proposed as an effective treatment for some forms of diabetes, and *in vitro* methods for β-cell generation are being extensively explored. A potential source of β-cells comes from fate conversion of exocrine pancreatic cells into the endocrine lineage, by overexpression of three regulators of pancreatic endocrine formation and β-cell identity, Ngn3, Pdx1 and MafA. Pancreatic ductal organoid cultures have recently been developed that can be expanded indefinitely, while maintaining the potential to differentiate into the endocrine lineage. Here, using mouse pancreatic ductal organoids, we see that co-expression of Ngn3, Pdx1 and MafA are required and sufficient to generate cells that express insulin and resemble β-cells transcriptome-wide. Efficiency of β-like cell generation can be significantly enhanced by preventing phosphorylation of Ngn3 protein and further augmented by conditions promoting differentiation. Taken together, our new findings underline the potential of ductal organoid cultures as a source material for generation of β-like cells and demonstrate that post-translational regulation of reprogramming factors can be exploited to enhance β-cell generation.

## Introduction

Pancreas function involves complex orchestration of exocrine and endocrine cell activities to maintain metabolic homeostasis, while a failure of glucose sensing and insulin production due to endocrine β-cell impairment underlies both Type 1 and some forms of Type 2 diabetes^1,2^. Hence, the huge public health burden of diabetes has led to intense investigation of pancreatic endocrine cell formation, with the hope that new ways will be found to generate β-cells *in vitro* or *in vivo* for cell replacement therapies and/or to support β-cell survival and function^2–7^.

Generation of new mammalian β-like cells *in vivo* has been achieved by transcription factor-mediated direct reprogramming of pancreatic acinar cells to endocrine cells using three transcription factors, Neurogenin3 (Ngn3 or Neurog3), Pdx1 and MafA^8,9^, which play a central role in endocrine cell development and in mature adult β-cells^10,11^. Moreover, β-like cells have been generated from cells of the gastrointestinal tract *in vivo* showing that different cell types are susceptible to this directed reprogramming approach^12^. *Ex vivo* generation of β-cells for potential cell replacement therapy has also been extensively explored using embryonic stem cells and a complex regime of fate altering growth factors^13–15^.

Pancreatic islet cells are specified within the developing ducts in embryogenesis, while islet neogenesis declines in adulthood^16–19^. Ductal cells isolated from the adult pancreas, however, contain a population of stem-like cells that grow *in vitro* as 3D ‘organoids’^20^. These cells also maintain a limited multi-lineage potential, as *in vivo* transplantation of ductal organoids allows some cells to adopt an endocrine fate^20^. The indefinite proliferative capacity of ductal organoids makes them an attractive potential source of endocrine, and particularly β-cells, for disease modelling and cell replacement, but for this potential to be realised we must achieve more efficient reprogramming of these cells to endocrine fate *in vitro*.

Ngn3 expression in ductal-derived cells is sufficient to turn on an array of downstream targets known to be associated with endocrine pancreas cell formation^21–23^, while co-expression of Ngn3, Pdx1 and MafA in 3D cultures of human pancreatic ducts has led to generation of β-like cells, albeit at low efficiency^22^. We have also recently seen that preventing Cdk-mediated phosphorylation of Ngn3 can potentiate endocrine differentiation in the pancreas *in vivo*^21,24^, and so post-translational modification of Ngn3 may play a role in the control of reprogramming efficiency *in vitro*. To further explore the transcription factor-mediated reprogramming capacity of mammalian pancreatic ductal cells we have investigated the transcriptional consequences of overexpressing Ngn3, Pdx1 and MafA singly, pair-wise or all together in ductal organoids. We find that all three factors are required and together are sufficient for conversion of mouse ductal organoid cells to β-like cells, while expression of only one or two of these transcription factors can induce other pancreatic hormones, but not insulin. We also find that substituting wild-type Ngn3 with a phospho-mutant version that cannot be phosphorylated by Cdks significantly enhances programming efficiency, which can be further augmented by transferring organoids to a medium lacking growth factors. Taken together, our findings demonstrate that transcription factor-mediated mammalian reprogramming of ductal organoids is a promising source of endocrine cells *in vitro*, while efficiency of β-like cell generation can be enhanced by regulating transcription factor post-translational modification and environmental cues.

## Results

### Reprogramming pancreatic organoids into distinct endocrine lineages

**Figure 1:**
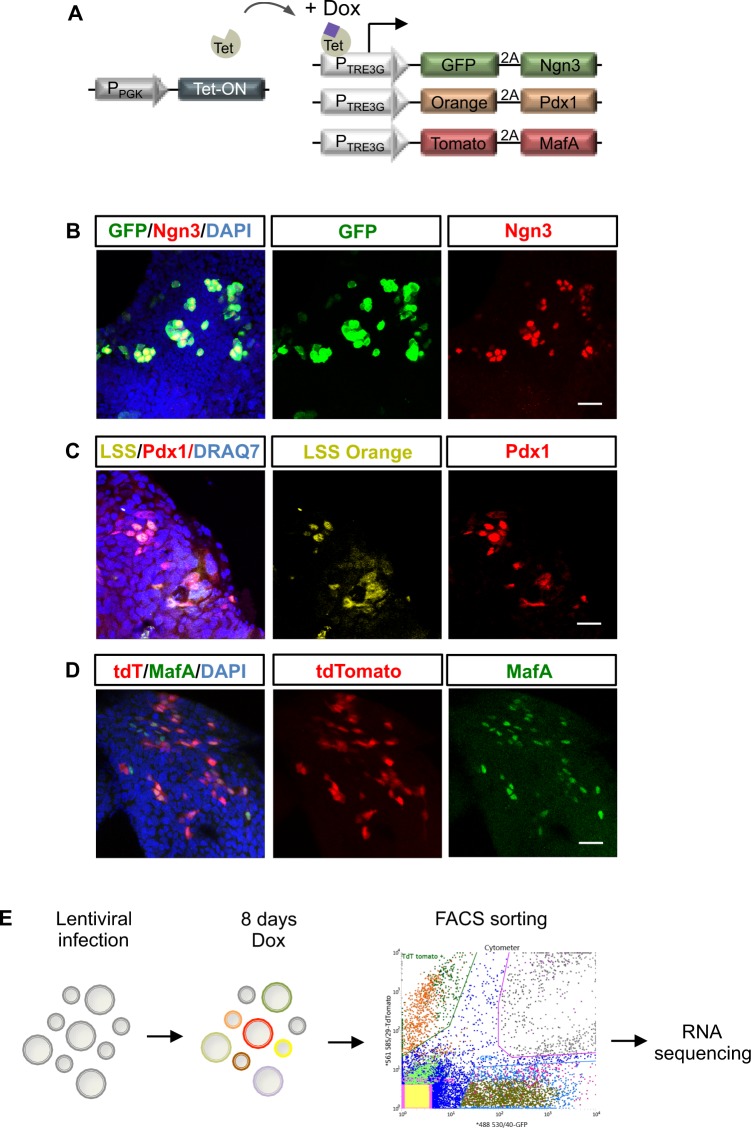
Expression of reprogramming transcription factors in pancreatic organoids infected with multiple vectors. (**A**) Schematic of the doxycycline inducible *multicolor* vector system. Immunostaining showing co-expression of GFP and Ngn3 (**B**); LSS-Orange and Pdx1 (**C**); Tomato and MafA (**D**). Nuclei are counterstained with DAPI (**B**,**D**) or DRAQ7 (**C**). Scale bar: B, C, D: 20 μm. (**E**) Experimental schematic. Pancreatic organoids were infected with viruses encoding GFP-Ngn3, LSS-Orange-Pdx1 and Tomato-MafA and expression induced with doxycycline for 8 days. Genome-wide RNA sequencing was performed on organoid cells sorted for the different fluorescent marker combinations.

**Figure 2:**
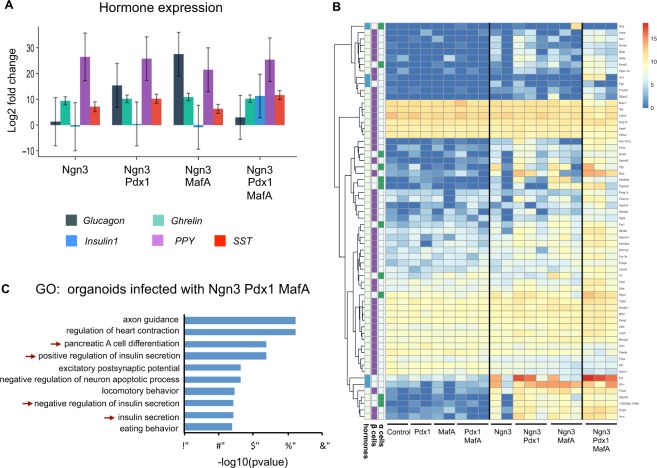
Reprogramming of pancreatic organoids into endocrine lineages. (**A**) Graph showing the log2 fold change expression of pancreatic hormones in cells expressing GFP-Ngn3 alone or in combination with Pdx1 and/or MafA, compared to control uninfected cells. Data represent average log2 fold change and error bars represent 95% confidence intervals of the mean. PPY: Pancreatic Polypeptide Y; Sst: Somatostatin. (**B**) Heatmap shows expression of α and β-cell genes^29^ that are significantly differentially regulated between control and Ngn3-Pdx1-MafA expression, alongside expression of endocrine homones. Biological replicates as indicated. (**C**) GO analysis of transcripts from pancreatic organoid cells co-infected with GFP-Ngn3, LSS-Orange-Pdx1, Tomato-MafA. Red arrows indicate gene ontology terms related to endocrine differentiation and insulin secretion.

To define the minimum set of transcription factors required to generate insulin-positive cells and other endocrine subtypes, we investigated the effects of lentivirus-mediated, doxycycline inducible expression of Ngn3, Pdx1 and MafA singly, in pairs or all together, in mouse ductal pancreatic organoids. To distinguish cells expressing one, two or three factors, we simultaneously infected cells with three separate vectors where each vector co-expresses a distinct fluorescent protein (Fig. 1A): GFP+ cells express Ngn3 (Fig. 1B), LSS-orange+ cells express Pdx1 (Fig. 1C) and Tomato cells express MafA (Fig. 1D). In addition, a vector carrying the doxycycline inducible transactivator was also co-infected (Fig. 1A). Organoids infected with multicoloured vectors were treated with doxycycline for 8 days to activate gene expression and were then FACS sorted (Fig. 2A), allowing us to detect and isolate cells that are expressing each of the transcription factors either individually or in each possible combination (Fig. 1E). Fifty cells of the same colour, and so expressing the same factor or factors, were pooled and subject to whole transcriptome sequencing as previously described^21^ (Fig. 1E). We first looked specifically at the expression of different pancreatic endocrine hormones to interrogate the requirement of specific transcription factor combinations for reprogramming towards different endocrine cell fates. Ngn3, when expressed alone in mouse ductal organoids, can upregulate transcripts of different hormones, including its direct target somatostatin (Sst) in the absence of ectopic Pdx1 or MafA, (Fig. 2A and^21^). However, Ngn3 alone is insufficient to activate α-cell-associated glucagon, which requires co-expression of either Ngn3 and Pdx1 or Ngn3 and MafA. Insulin transcripts were only detected in cells co-expressing Ngn3, Pdx1 and MafA, indicating more stringent transcriptional requirements for insulin expression compared to other hormones (Fig. 2A). We then looked more closely at transcriptome-wide changes in α and β cell genes in infected organoid cells. Expression of Ngn3, Pdx1 and MafA, either alone or in combinations, resulted in accumulation of a set of transcripts typically expressed in α and β cells, with maximal activation when all the three transcription factors are transduced (Fig. 2B).

Gene ontology (GO) analysis demonstrated significant upregulation of transcripts associated with pancreatic endocrine cell differentiation and insulin secretion in organoid co-expressing Ngn3, Pdx1 and MafA (Fig. 2C). Additional GO terms that were significantly enriched included those associated with neuron behaviour. This may reflect the prominent role of Ngn3 in many aspects of the neuronal programme, some of which are shared with pancreatic endocrine cells^25,26^. It was interesting to note changes in genes associated with eating behaviour; Ngn3 is involved in specification of pro-opiomelanocortin (POMC) neurons that are known to control appetite^27^. It is not clear the extent to which pancreatic and neuronal pathways share common genes, resulting in inappropriate GO assignment of neuronal categories or whether upregulation of neural pathways represents programming of pancreatic ducts down an inappropriate neuronal trajectory.

Altogether our data indicate that different transcription factor combinations strongly influence distinct endocrine hormone expression profiles when expressed in pancreatic ductal organoid cells and that only the co-expression of Ngn3, Pdx1 and MafA can drive cells down the β-cell lineage.

### Ngn3 protein phosphorylation and reprogramming to endocrine fates

**Figure 3:**
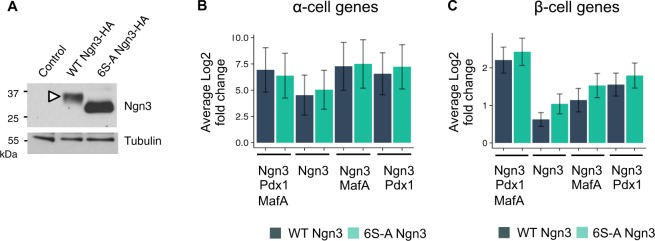
Ngn3 protein phosphorylation and endocrine reprogramming. (**A**) Western blot showing expression and phosphorylation of HA-tagged Ngn3 in pancreatic organoids. Tubulin is used as loading control. Full length western blots are provided in Supplementary Fig. S1A and S1B. (**B**,**C**) Expression of pools of 26 α-cell-enriched genes (**B**) and 151 β-cell enriched genes (**C**) in pancreatic organoids infected with multiple coloured vectors expressing combinations of Ngn3, Pdx1 and MafA, as labelled (graph bars represent pools of 26 α-cell genes and 151 β-cell genes, described in Dataset_S02 and Dataset_S03 of Supporting Information in Xin *et al*., PNAS 2016^29^). Data represent average log2 fold change and error bars represent 95% confidence intervals of the mean.

Previous work from our group and others has shown that the ability of Ngn3 to drive endocrine differentiation in the pancreas is restrained by its multi-site phosphorylation by cyclin-dependent kinases (Cdks)^21,24,28^. Western blotting demonstrated that wild-type Ngn3 isolated from infected ductal organoids migrates more slowly on SDS PAGE than phospho-mutant Ngn3, consistent with Ngn3 phosphorylation in organoids (Fig. 3A and Supplementary Fig. S1). We therefore set out to test whether a phospho-mutant form of Ngn3, which cannot be phosphorylated as Cdk target serine sites have been mutated to alanine (6S-A Ngn3)^21^, may be better at driving endocrine differentiation in ductal organoids, compared to wild-type Ngn3. To this end, we used our three vector system to undertake a genome-wide comparison of transcripts associated with endocrine formation in organoid cells expressing wild-type or phosphomutant Ngn3, with or without Pdx1 and/or MafA. In particular, we focused on gene sets associated with α- and β-cell identity that have been recently identified through single cell analysis of mouse pancreatic islets^29^.

Transcriptome-wide analysis revealed that wild-type Ngn3 expression alone is sufficient to enhance expression of a set of α-cell genes identified in^29^ (Fig. 3B), even though we had previously seen that it was insufficient to upregulate glucagon, the hormone associated with α-cell identity (Fig. 2A). α-cell transcripts induced by Ngn3 are not significantly augmented by addition of Pdx1, MafA or both together. Moreover, no difference in α-gene expression was observed when phospho-mutant Ngn3 was substituted for WT Ngn3. We next investigated upregulation of a pool of transcripts associated with β-cell identity^29^ in the presence of wild-type or phospho-mutant Ngn3. Wild-type Ngn3 expression upregulated β-cell genes on its own and in combination with Pdx1 or MafA, but consistent with the requirement for all three transcription factors to drive insulin expression, all three are required for the greatest up-regulation of β-cell-associated transcripts (Fig. 3C). When phospho-mutant Ngn3 was substituted for wild-type Ngn3, we noted that there was a trend towards higher expression of β-cell enriched genes under all conditions (Fig. 3C). However, as statistical significance was not reached, it was not clear whether this represented a subtle but genuine enhanced function of phospho-mutant Ngn3 in activation of β-cell targets compared to the wild-type protein or not.

**Figure 4:**
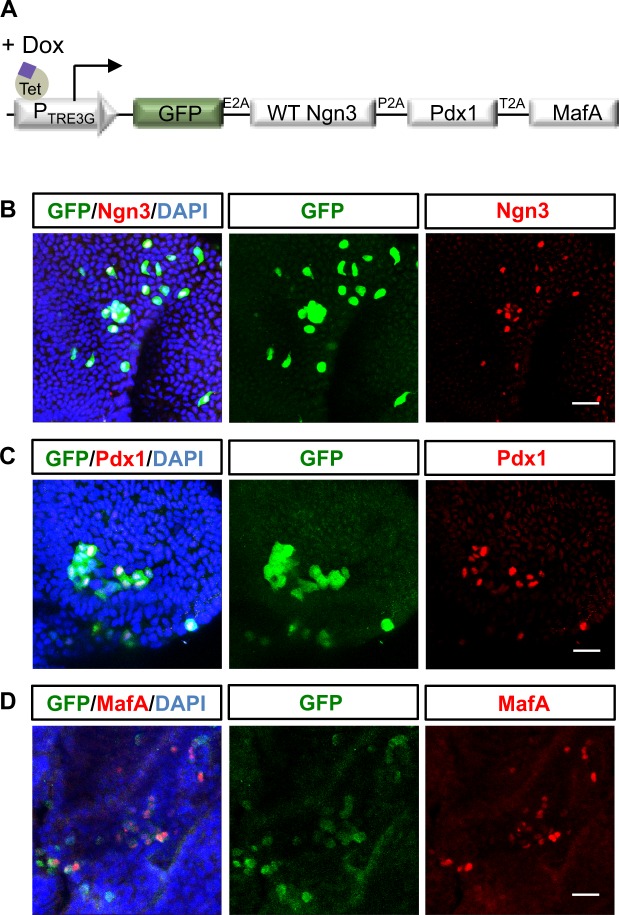
Expression of reprogramming transcription factors in pancreatic organoids infected with a multicistronic vector. (**A**) Scheme of the *multicistronic* vector system. E2A, P2A and T2A are cleavage peptides. (**B**–**D**) Immunostaining showing co-expression of GFP and Ngn3 (**B**), Pdx1 (**C**) or MafA (**D**). Nuclei are counterstained with DAPI, Scale bar: 20 μm.

Our three-vector system can be used to sort cells expressing different combinations of transcription factors, but it cannot ensure that all factors are equally expressed in all the 50 pooled cells used for transcriptome analysis. This could lead to unhelpful cell to cell variations in amounts of different vectors, which could be masking real effects that might be observed if reprogramming factors were expressed at equal levels. As maximal expression of β-cell transcripts was achieved by co-expression of Ngn3, Pdx1 and MafA, we then moved to a multi-cistronic vector system where wild-type or phospho-mutant Ngn3 were co-expressed as a single transcript, but where genes are separated by a 2A peptide sequence allowing cleavage into individual proteins during translation, alongside GFP to act as a tracer of infected cells (Fig. 4A)^30^. Antibody staining for Ngn3, Pdx1 and MafA showed that all 3 proteins were efficiently expressed in GFP positive cells when using this system (Fig. 4B–D).

### Un(der)phosphorylated Ngn3 enhances β-like cell conversion efficiency

**Figure 5:**
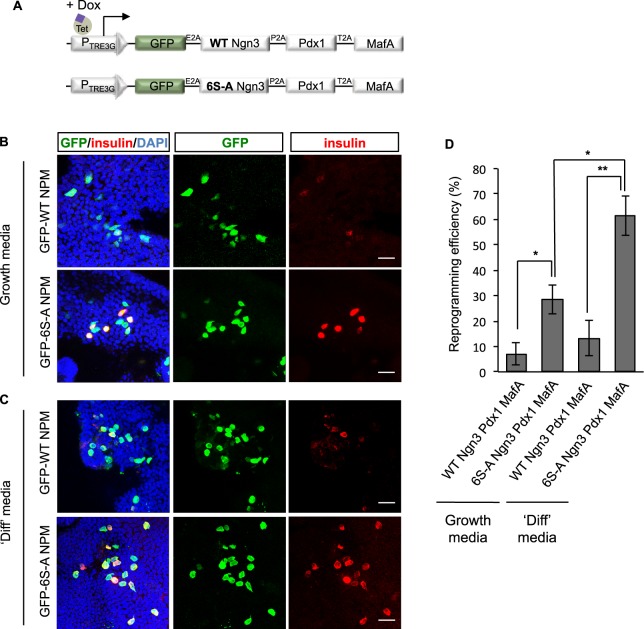
Un(der)phosphorylated Ngn3 enhances conversion to β-like cells. (**A**) Schematic of the multicistronic vectors expressing wild-type Ngn3 or phosphomutant (6S-A) Ngn3. (**B**–**D**) Analysis of cell fate conversion. Insulin expression 8 days after doxycycline induction of WT or 6S-A Ngn3-Pdx1-MafA (NPM) in either growth media (**B**) or differentiation (‘diff’) media, (**C**). (**D**) Efficiency of conversion to insulin positive β-like cells under conditions as labelled, expressed as the percentage of insulin+ cells over GFP+ infected cells. Data represent mean ± sem n = 3 *p < 0.05, **p < 0.01. Scale bars: 20 μm.

Using our multi-cistronic vector system, we next compared the efficiency of reprogramming to insulin-expressing β-like cells 8 days after induction of either wild-type or phospho-mutant Ngn3 alongside Pdx1, MafA and GFP as a tracer (Fig. 5A). To detect reprogramming of individual cells, we performed insulin immunostaining of infected cells. WT Ngn3/Pdx1/MafA expression resulted in 7 ± 4.5% of infected cells expressing sufficient insulin to be detected by antibody staining, while substituting phospho-mutant Ngn3 increased reprogramming efficiency to 28.3 ± 5.6% (Fig. 5B–D), an almost 4-fold increase in insulin-positive cells. Thus, preventing phosphorylation of Ngn3 on serine-proline cdk target sites significantly enhances its ability to drive reprogramming of pancreatic ductal organoid cells to insulin expressing β-like cells.

Removal of growth factors inhibits cell proliferation and enhances endocrine differentiation of pancreatic progenitors^31^. This may act mechanistically by inhibiting cdk-dependent phosphorylation of Ngn3^21,28^ and/or via additional mechanisms that potentiate differentiation. We next tested whether growth factor removal is able to further enhance insulin expression. We found that transferring organoids into a medium promoting differentiation (without EGF and R-Spondin and with the addition of the Wnt inhibitor, IWP-2) for the last two days of culture, resulted in an approximate doubling of the percentage of insulin-expressing cells (Fig. 5C,D). In particular, in the presence of phospho-mutant Ngn3, the percentage of insulin-positive cells rises to 61.7 ± 18.8% (Fig. 5D). This further augmentation of insulin positive cells by replacing WT Ngn3 with a phosphomutant form of the protein indicates that growth factor withdrawal has additional roles in enhancing endocrine differentiation beyond control of cdk-dependent phosphorylation of Ngn3. This rate of conversion of *in vitro* expanded ductal cells to β-like insulin-expressing cells is, to our knowledge, one the highest rates of conversion, if compared for example with previous reprogramming of human 3D ductal culture that reached an efficiency of approximately 7–11%^22^.

### Analysis of hormone co-expression in induced β-like cells

**Figure 6:**
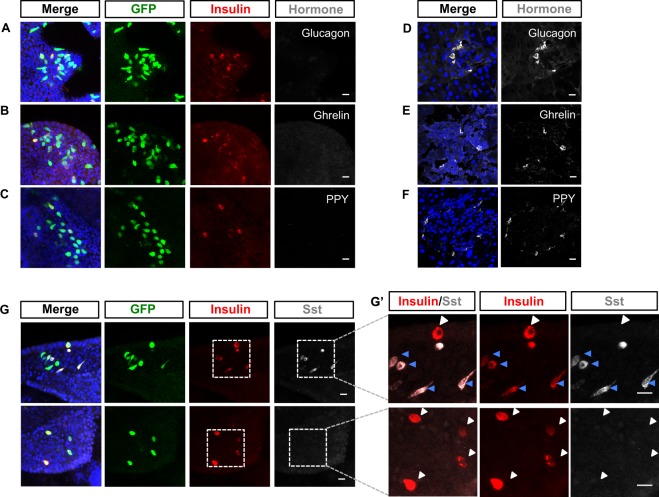
Co-localization of insulin with other hormones in reprogrammed endocrine cells. (**A**–**C**) Pancreatic organoids were infected with the multicistronic vector expressing GFP-6S-ANgn3-Pdx1-MafA. Immunostaining for insulin (red) and Glucagon, Ghrelin, or PPY (grey) was undertaken 8 days after doxycycline treatment. Hormone expression in pancreatic islets is used as a positive control for immunostaining in organoids (**D**–**F**, as labelled). (**G**) Immunostaining for both insulin (red) and Somatostatin (grey) in pancreatic organoids infected with multicistronic vectors as in A–C. **(G’**) Magnification of images in G, to illustrate both Sst/insulin and monohormonal insulin expressing cells (insulin, red, and somatostatin, grey). White arrows show monohormonal insulin positive cells and blue arrows indicate multi-hormonal insulin and Sst positive cells. Nuclei in A–G are counterstained with DAPI. Scale bars: 20 μm.

Mature pancreatic endocrine α-, β- and δ-cells typically express either glucagon, insulin or somatostatin alone, while expression of more than one hormone is a characteristic of immature endocrine cells^32–34^. Moreover, a frequent outcome in embryonic stem cell mediated reprogramming protocols to generate β-cells is that cells co-express multiple hormones^35–37^. Our genome-wide transcriptomic analysis indicates that several pancreatic hormones are expressed by reprogrammed ductal cells, but pooling of 50 cells for transcriptome analysis precludes identifying whether individual cells express multiple hormones. To determine whether transcription factor-mediated reprogramming generated cells that express insulin alone or in combination with other hormones, we took organoids expressing phospho-mutant Ngn3, Pdx1 and MafA with GFP from our multi-cistronic vector 8 days post induction and stained for insulin alongside Glucagon, Ghrelin, Pancreatic polypeptide-Y (PPY) and Sst (Fig. 6), using antibodies validated for hormone detection in pancreatic islets (Fig. 6D–F).

Confirming our transcriptome analyses (Fig. 2A), insulin was clearly detected in cells expressing mutant Ngn3, Pdx1 and MafA (Fig. 6). However, we did not observe any insulin+ cells that co-express Glucagon, Ghrelin or PPY (Fig. 6A–C and Supplementary Fig. S2A–C). We also co-stained for insulin alongside Sst and found that 33.5 ± 5.9% of cells co-expressed the two hormones (blue arrows in Fig. 6G-G’ and Supplementary Fig. S2D,E). However, the majority of the reprogrammed endocrine cells were mono-hormonal for insulin (white arrows in Fig. 6G-G’ and Supplementary Fig. S2E), thus resembling more mature β-cells.

**Figure 7:**
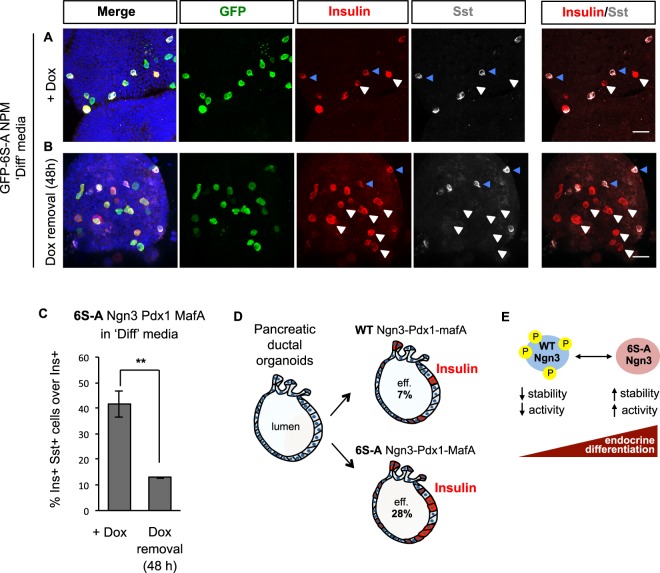
Co-expression of insulin and somatostatin in reprogrammed pancreatic organoid cells in conditions promoting differentiation. (**A**,**B**) Representative images of pancreatic organoids infected with multicistronic vectors expressing GFP-6S-ANgn3-Pdx1-MafA (NPM), transferred to differentiation medium for the last two days of culture, with (**A**) or without (**B**) doxycycline. Cells are immunostained for insulin (red) and somatostatin (Sst, grey). White arrows show mono-hormonal insulin positive cells and blue arrows indicate multi-hormonal insulin and Sst positive cells. Nuclei are counterstained with DAPI. Scale bars: 20 μm. (**C**) Graph showing quantification of the percentage of insulin + cells that also co-express Sst. Data represent mean ± sem n = 3 **p < 0.01. (**D**) Schematic summarizing major findings. Pancreatic ductal organoids infected with Ngn3, Pdx1 and MafA generate insulin positive cells at low efficiency (eff.). Substitution of wild type (WT) with phosphomutant (6S-A) Ngn3 enhances reprogramming efficiency. (**E**) Model of phosphorylation-mediated regulation of Ngn3. Inhibition of Ngn3 phosphorylation increases protein stability and Ngn3 activity and enhances differentiation of endocrine cells^21,28,39^.

Since mixed hormone identity of *in vitro* generated endocrine cells would limit the use of such cells therapeutically, we asked whether we could reduce or eliminate Sst co-expression by further promoting organoid differentiation. We therefore quantified hormone co-localization after transferring organoids into the differentiation medium described above (Fig. 5C,D) for the final two days of culture, but found a similar proportion of insulin and Sst co-positive cells (41.4 ± 5.1%) as when organoids were maintained in growing conditions (33.5 ± 5.9%) (Fig. 7A–C). We reasoned that continued expression of Ngn3 that is a direct activator of the *Sst* gene might lead to sustained Sst expression. Therefore, we removed doxycycline from the differentiation media to downregulate the expression of the reprogramming transcription factors for the last two days of culture and observed the percentage of insulin and Sst co-expressing cells dropping from 41.4 ± 5.1% to 12.9 ± 0.32% (Fig. 7B,C).

Altogether, our results show that pancreatic organoids can be reprogrammed into β-like cells with high conversion efficiency, through the concerted regulation of Ngn3 activity and environmental cues.

## Discussion

*In vitro* generation of β-cells for replacement therapies in diabetes could improve the quality of life of millions of people and massively reduce the burden of its long-term complications. Several laboratories have developed protocols to differentiate *in vitro* human embryonic stem cells down the β-cell lineage. While some of the most recent protocols achieve functional maturation of β-cells, reproducibility and scalability are still major issues when bringing this into the clinic, as is generation of cells with a mono-hormonal identity^4,13,15,35–37^.

Another potential source of β-cells comes from the trans-differentiation of other cell types, most notably cells of the exocrine pancreas. Adenoviral delivery of Ngn3, Pdx1 and MafA *in vivo* has been shown to reprogram acinar cells and other cells of the gastrointestinal tract into β-like cells that can ameliorate hyperglycaemia in an experimental animal model of diabetes^8,9,12^. Endocrine cells arise during development by delamination from the emerging ducts^17,18^ and so ducts might offer a potentially more physiologically relevant source of cells for efficient reprogramming to β-cells. Indeed, recent new methods have been developed to isolate and grow pancreatic ducts in 3D culture^20,38^, fuelling interest in the reprogramming capacity of these cells. Here we have interrogated the reprogramming capacity of mouse ductal organoids in response to transcriptional regulators that control endocrine cell developmental and mature β-cell function, with the aim of promoting generation of insulin-expressing cells.

Using lentiviral-mediated overexpression, we see that expression of the developmental transcription factor Ngn3 induces pancreatic hormone expression in ductal organoids cells, but the addition of Pdx1 and MafA is required specifically for insulin expression. Consistent with previous studies however^22^, wild-type Ngn3, Pdx1 and MafA co-expression results in only a small percentage of reprogrammed cells expressing insulin. Here we show that preventing Ngn3 phosphorylation at cdk targeted sites results in an almost 4-fold increase in fate reprogramming (Fig. 7D). Indeed, the percentage of cells expressing insulin we achieved is considerably higher than what was observed in other similar reprogramming contexts^22^, indicating that manipulating phosphorylation of Ngn3 may be a useful way to enhance β-cell reprogramming protocols. The enhanced ability of phosphomutant Ngn3, in combination with MafA and Pdx1, to promote β-cell gene expression is likely to be due at least in part to increased stability and DNA binding^21,28^. Phosphorylation of Ngn3 on Serine-Proline sites has been previously shown *in vivo* to be required for recruitment of E3 ubiquitin ligases leading to protein degradation^39^, while chromatin association of a number of transcription factors has been shown to be regulated by multisite phosphorylation^40,41^ (Fig. 7E).

As Ngn3 is known to be targeted by cdks^21,28^, we reasoned that removing growth factors from culture medium may potentiate Ngn3 function by inhibiting the cell cycle resulting in dephopshorylation of Ngn3. However, it is of note that we observed an additional doubling of insulin-expressing cells after growth factor withdrawal in the presence of phospho-mutant Ngn3. This indicates that growth factors suppress insulin expression by additional mechanisms as well as promoting phosphorylation of Ngn3 protein and thereby restricting its ability to drive the differentiation programme. Thus, the incorporation of phosphomutant Ngn3 and/or cdk inhibitors alongside further manipulation of the growth factor environment could be explored to further increase efficiency of β-cell generation in other *in vitro* reprogramming protocols^28,31,42^.

β-like cells we generated from directed reprogramming of organoids displayed a transcriptome-wide profile consistent with β-cell identity and expressed insulin. However, how closely they resemble mature adult β-cells will need to be explored more fully; our attempts to measure glucose-simulated insulin secretion were inconclusive, as these small-scale organoid studies did not provide a large enough number of cells for reliable analysis by standard methods^43^. β-like cells generated at low efficiency from human ductal 3D cultures show evidence of insulin storage and secretion^22^, and this indicates that enhancing reprogramming by inhibiting phosphorylation of Ngn3 and removal of growth factors may more efficiently generate functional β-cells. Assessing glucose responsiveness will be a crucial next step for determining whether β-like cells generated form ductal organoids are functional.

Normal β-cells are mono-hormonal, while multi-hormone expression has been observed in diseased state^44^ and in other programming/reprogramming regimes^35–37^ and is likely to be detrimental for endocrine cell function. Here we show that (please make mono-hormonality lower-case) Mono-hormonality of endocrine cells generated in organoids is significantly improved by downregulating the expression of reprogramming factors for the last two days of organoid culture (Fig. 7C,D). Therefore, both the nature and timing of expression of reprogramming factors clearly influences the efficiency of generation of hormone-expressing cells in pancreatic organoids as well as mono/poly-hormonal identity.

The best reprogramming strategy should activate an endogenous transcription factor network capable of maintaining the new cell identity, even when the exogenous reprogramming transcription factors are removed. While a stable new β-cell state has been achieved from conversion of acinar cells^8^, intestinal cells, in a similar setting, fail to maintain insulin expression upon removal of the exogenous factors^12^. Therefore, future studies will need to address genetic and epigenetic events that lock cells into their specific cell identity.

The work described above has exploited a mechanistic understanding of Ngn3 post-translational regulation gained from our previous studies of endocrine pancreatic development in the embryo. It illustrates well how understanding the basic mechanisms underlying normal cell and tissue development can be exploited to enhance cellular reprogramming for regenerative applications.

## Materials and Methods

### Pancreatic organoid culture

Pancreatic organoids were derived and cultured as previously described^20,45,46^. Briefly, adult pancreatic tissue was digested for 45–60 minutes at 37 °C with the collagenase/dispase dissociation Medium (1% in DMEM media (Gibco), supplemented with 1% FBS (Gibco) and Collagenase type XI 0.012% (w/v) (Sigma), dispase 0.012% (w/v) (Gibco)). Single ducts were manually picked and seeded in Matrigel (BD Bioscience). Ducts were cultured in AdDMEM/F12 (Invitrogen) supplemented with N2 and B27 (Lifesciences), 1.25 mM N-Acetylcysteine (Sigma), 10 nM gastrin (Sigma) and the growth factors: 50 ng/ml EGF (Peprotech), 5% RSPO1-conditioned media, 5% Noggin-conditioned media (in-house prepared), 100 ng/ml FGF10 (Peprotech) and 10 mM Nicotinamide (Sigma). Differentiation medium consisted in removal of EGF, RSPO1-conditioned media and addition of the Wnt Inhibitor IWP-2 (0,25 μM, Stemgent^47^) for the last 2 days of induction, with or without doxycycline, as indicated.

### Plasmid vectors

The coding sequences for mouse Ngn3, Pdx1 and MafA (Addgene: #19412) were cloned into pWPT-eGFP-E2A (for Ngn3), pWPT-LSSmOrange-E2A (for Pdx1) and pWPT-dTomato-E2A (for MafA) and subcloned in the doxycycline inducible lentivirus pLVX_TRE3G vector (Clontech). Ngn3, Pdx1 and MafA were cloned in the multicistronic vector pWPT-eGFP-Poly3TF and subcloned in the lentivirus pLVX_TRE3G vector (Clontech). The doxycycline inducible Tet-On® 3G transactivator (Clontech) was cloned under PGK (phosphoglycerate kinase) promoter. Multicolor and multicistronic lentiviral backbone vectors were kind gift from Dr. Cedric Ghevaert and Dr. Thomas Moreau^30^. Phosphomutant Ngn3 was prepared by site directed mutagenesis (QuickChange II Site-Directed Mutagenesis Kit, Stratagene) on serines S14, S38, S160, S174, S183 and S199, as described in^21^.

### Pancreatic organoid infection

Pancreatic organoids infection was performed as described in^21^. Viruses were produced in HEK293T cells and used at multiplicity of infection of 10 for the transgene or 20 for the transctivator Tet-On® 3G. Organoids were dissociated into small clusters, incubated with culture media containing the viruses, 8 μg/ml polybrene (Sigma), 10 μM ROCKi (Sigma) and span for 1 h at 300 × G at room temperature. After the spin, infected organoids were incubated in a cell culture incubator at 37 °C for 5–6 hours before plating in matrigel with fresh media supplemented with ROCKi.

### RNA sequencing of infected pancreatic organoids

Pancreatic organoids were infected with multicolour vectors and treated with doxycycline (1 μg/μl) for 8 days. Organoids were then dissociated into single cells and pools of 50 cells for each colour combination were FACS sorted and collected into a 96 well plate containing lysis buffer. Library preparation for RNA sequencing was performed with Illumina Nextera XT DNA preparation kit, following the protocol for single cell RNA sequencing described in^48,49^. Pooled libraries were sequenced using the Illumina HiSeq 4000 system (single-end 50 bp reads).

### Western Blotting

Pancreatic organoids were infected with GFP-WT-Ngn3-HA or GFP-6S-A-Ngn3-HA and treated with doxycycline (1 μg/μl) for 2 days. Infected GFP+ cells were FACS sorted and lysed in RIPA-like lysis buffer (50 mM Tris-HCl, pH 8; 150 mM NaCl; 0.5% NP40 Igepal; 10% Glycerol, supplemented with protease and phosphatase inhibitor cocktails (Roche; Calbiochem)). Proteins were separated on SDS-PAGE and immmunoblotted for rat anti-HA-Peroxidase (1:1000; Roche) and mouse anti-tubulin (1:1000, Sigma).

### Immunochemistry

Pancreatic organoids were extracted from Matrigel with Cell Recovery solution (Sigma), fixed in 4% paraformaldehyde (PFA) for 20 minutes at 4 °C and immunochemistry performed in suspension. Primary antibodies: insulin (guinea pig, 1:200, Abcam ab7842), glucagon (mouse, 1:200, Abcam ab10988), Somatostatin (rabbit, 1:200, Dako A0566), PPY (goat, 1:200, Abcam ab77192), ghrelin (rat, 1:50, R&D System MAB8200-SP), GFP (chicken, 1:600, Abcam ab13970), dsRed (mouse, 1:100, Clontech 632393), Ngn3 (goat, 1:100 Santa Cruz sc-13793, discontinued), Pdx1 (guinea pig, 1:200, Abcam ab47308); MafA (rabbit, 1:200, Cambridge Bioscience IHC-00352). MafA required Antigen retrieval for 10 minutes at 90 C in citrate pH6 prior to primary antibody incubation.

### Bioinformatic analysis of RNA sequencing

Read alignment was performed using G-SNAP 30 and mapped reads were assigned to Ensembl genes (release 81) by HTSeq. Poor quality samples were identified by three metrics: (1) the proportion of reads aligned to spike ins, (2) number of endogenous reads and (3) number of features with read more than zero. Cells were filtered with (1) <25% reads aligned to spike ins, (2) >1000000 endogenous reads and (3) >10000 detected features. We only considered genes that were detected in at least five samples (including technical and biological repeats), with variance >1e-3. Based on this, five samples were not considered for downstream analysis. Technical repeats were merged and DESeq 2 package version 1.20 with betaPrior = F and no further shrinkage of log2 fold changes was used for differential expression analysis^50^. Heatmap shows log expression values after size factor normalisation.

## Electronic supplementary material

Supplementary Information

## Data Availability

All sequencing data are deposited in GEO: GSE114838. All data generated or analysed during this study are included in this article and its supplementary material.
