# Supplementary material for: Neurogenin3 phosphorylation controls reprogramming efficiency of pancreatic ductal organoids into endocrine cells
Source: Sci Rep. 2018 Oct 18;8:15374. doi: 10.1038/s41598-018-33838-5 (PMC6193982; doi:10.1038/s41598-018-33838-5)
Supplement: Supplementary file 1 — Supplementary Information [file 41598_2018_33838_MOESM1_ESM.pdf]

Supplementary Information

Neurogenin3 phosphorylation controls reprogramming efficiency of pancreatic ductal organoids into endocrine cells

Roberta Azzarelli[1,2,3], Steffen Rulands[4,5], Sonia Nestorowa[2,6], John Davies[1,2], Sara Campinoti[7,8], Sebastien Gillotin[1,2], Paola Bonfanti[7,8], Berthold Göttgens[2,6], Meritxell Huch[9], Benjamin Simons[2,3,9], Anna Philpott[1,2]

Supplementary Fig. S1 (relative to Fig. 3)

A

B

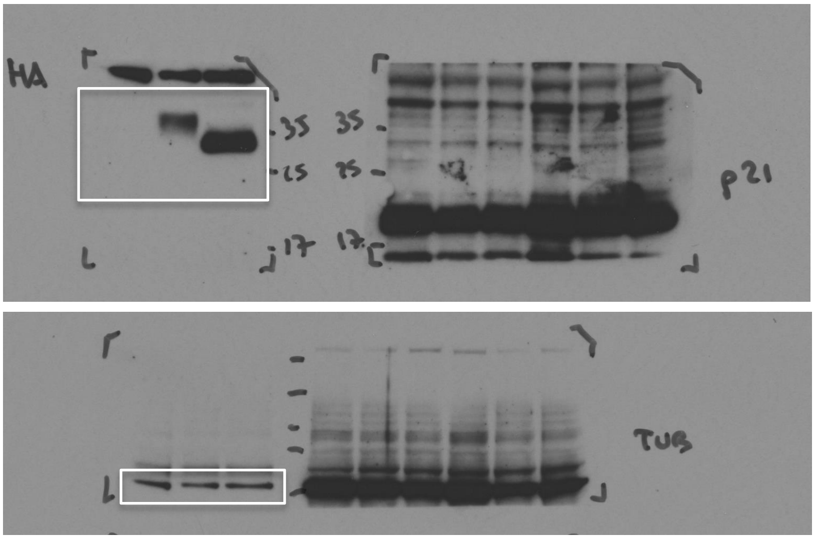

Supplementary Figure S1. Full length Western Blot gels. (**A**) Full length gel for Fig. 3A. White rectangle indicates HA-tagged Ngn3. (**B**) Full length gel for Fig. 3A. White rectangle indicates tubulin. Other samples marked for either p21 (A) or tubulin (B) are present in the same gel.

Supplementary Fig. S2 (relative to Fig. 6)

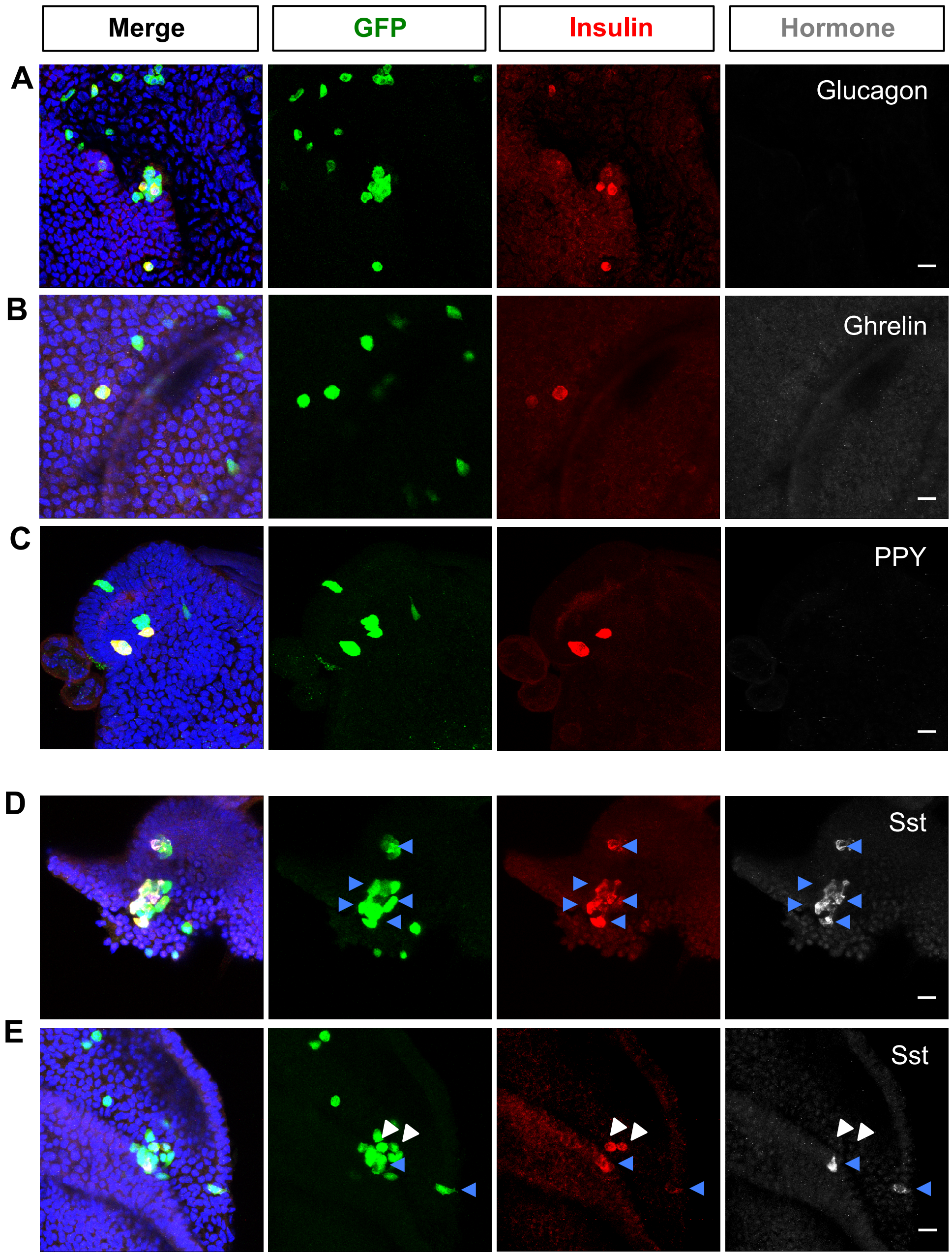

Supplementary Figure S2. Hormone co-expression in induced β-like cells. (**A-E**) Additional representative images of pancreatic organoids infected with multicistronic vectors expressing GFP-6S-ANgn3-Pdx1-MafA, 8 days after doxycycline treatment and immunostained for insulin (red) and glucagon, ghrelin, PPY or somatostatin (grey). White arrows show monohormonal insulin positive cells (**D**) and blue arrows indicate multi-hormonal insulin and Sst positive cells (**E**). Nuclei are counterstained with DAPI. Scale bars: 20 µm.
